# Resolvin D1 alleviates apoptosis triggered by endoplasmic reticulum stress in IPEC-J2 cells

**DOI:** 10.1186/s12917-023-03820-z

**Published:** 2024-04-01

**Authors:** Siyuan Zhu, Jingbo Liu, Qi Wang, Yong Yang, Lei Du, Xiaoyu Qiu, Renli Qi, Jing Wang

**Affiliations:** 1 Chongqing Academy of Animal Sciences, Chongqing, 402460 P. R. China; 2 School of Life Science and Engineering, Southwest University of Science and Technology, Mianyang, 621010 P. R. China; 3 State Key Laboratory of Animal Nutrition, College of Animal Science and Technology, China Agricultural University, Beijing, 100193 P. R. China

**Keywords:** Resolvin D1, Endoplasmic reticulum stress, Apoptosis, Tunicamycin, IPEC-J2 cells

## Abstract

**Background:**

Resolvin D1 (RvD1), a specialized pro-resolving lipid mediator (SPM), is derived from docosahexaenoic acid (DHA). It plays a key role in actively resolving inflammatory responses, which further reduces small intestinal damage. However, its regulation of the apoptosis triggered by endoplasmic reticulum (ER) stress in intestinal epithelial cells is still poorly understood. The intestinal porcine epithelial cells (IPEC-J2) were stimulated with tunicamycin to screen an optimal stimulation time and concentration to establish an ER stress model. Meanwhile, RvD1 (0, 1, 10, 20, and 50 nM) cytotoxicity and its impact on cell viability and the effective concentration for reducing ER stress and apoptosis were determined. Finally, the effects of RvD1 on ER stress and associated apoptosis were furtherly explored by flow cytometry analysis, AO/EB staining, RT-qPCR, and western blotting.

**Results:**

The ER stress model of IPEC-J2 cells was successfully built by stimulating the cells with 1 µg/mL tunicamycin for 9 h. Certainly, the increased apoptosis and cell viability inhibition also appeared under the ER stress condition. RvD1 had no cytotoxicity, and its concentration of 1 nM significantly decreased cell viability inhibition (*p*= 0.0154) and the total apoptosis rate of the cells from 14.13 to 10.00% (*p*= 0.0000). RvD1 at the concentration of 1 nM also significantly reduced the expression of glucose-regulated protein 78 (*GRP-78*, an ER stress marker gene) (*p*= 0.0000) and pro-apoptotic gene *Caspase-3* (*p*= 0.0368) and promoted the expression of B cell lymphoma 2 (*Bcl-2*, an anti-apoptotic gene)(*p*= 0.0008).

**Conclusions:**

Collectively, the results shed light on the potential of RvD1 for alleviating apoptosis triggered by ER stress, which may indicate an essential role of RvD1 in maintaining intestinal health and homeostasis.

**Supplementary Information:**

The online version contains supplementary material available at 10.1186/s12917-023-03820-z.

## Background

Intestine plays a critical role in various biological functions, including nutrient digestion and absorption, immune regulation, microbe defense and hormone secretion. To fulfill these functions, intestinal epithelial cells have a well-developed endoplasmic reticulum (ER). However, various physiological and pathological factors, including nutritional insufficiencies, pathogen infiltration, and bacterial infections, have the propensity to readily trigger endoplasmic reticulum (ER) stress in the epithelial cells of porcine intestines. This, in turn, culminates in cellular apoptosis and subsequent impairment of the intestinal barrier. Consequently, nutrient absorption is compromised, immune defense against pathogens is weakened, and the overall health of pigs is ultimately compromised [1–5]. The unfolded and misfolded proteins increase in ER lumen during the occurrence and development of ER stress, which causes an increase in glucose-regulated protein 78 (GRP-78) [6], a molecular chaperone, that participates in the correction of misfolded proteins and avoids the transport of unfolded and misfolded proteins. Thus, *GRP-78* is often regarded as an ER stress marker gene [7–9].

At the early developmental stage, ER stress initiates unfolded protein response (UPR) that protects cells from stress and re-establishes cellular homeostasis [10, 11]. However, prolonged ER stress triggers cell apoptosis [10, 12], due to various UPR-induced mechanisms can’t successfully alleviate ER stress causing that ER function fails to return to normal [13, 14]. Tunicamycin is usually used to build ER stress, which in turn triggers cell apoptosis [15–17]. The occurrence of apoptosis mainly relies on the activation of intrinsic and/or extrinsic apoptosis pathways [10, 18]. Both pathways activate the apoptotic effector Caspase-3 [10, 19, 20]. B cell lymphoma 2 (Bcl-2)-associated X protein (Bax), a pro-apoptotic protein, localizes at ER membrane [14], once activated, it oligomerizes to initiate intrinsic apoptosis [21]. In addition to its central role in intrinsic apoptosis pathway, anti-apoptotic protein Bcl-2 also localizes at ER membrane and plays a protective role against ER stress [14]. Mutual antagonism between Bax and Bcl-2 regulates intrinsic apoptosis [22]. To sum up, the protein expression of apoptotic markers, for example, Caspase-3, Bax, and Bcl-2, is broadly measured as conclusion basis of occurring apoptosis [23].

Resolvins, the newly discovered specialized pro-resolving lipid mediators (SPMs) [24], are derived from ω-3 polyunsaturated fatty acids (PUFA) [25, 26]. They are classified with the E series (Resolvin E, RvE) and D series (Resolvin D, RvD) according to distinct structural forms [26]. The D series play a key role in actively resolving acute inflammatory responses [24, 26]. Docosahexaenoic acid (DHA), an important ω-3 PUFA, can be ingested from food and conversed to RvD1 in animal body [27]. Research has shown that RvD1 prevents intestinal mucosal damage by reducing the gene expression levels of interleukin-1β, tumor necrosis factor-α, and CXCL1/keratinocyte chemoattractant[28].RvD1 increases the clearance of bacteria and white blood cells mediated by macrophages in mouse lungs, thereby reducing neutrophil infiltration and lung histopathological manifestations in mice [29].RvD1 inhibits the expression of inflammatory cytokines IL-6, IL-1, and TNF-α in muscle cells, reducing the inflammatory levels in muscle cells [30].Additionally, studies have also found that RvD1 alleviates hepatic steatosis and hepatocyte apoptosis through the JNK-mediated pathway [26]. RvD1 exerts its anti-inflammatory and anti-apoptotic effects in myocardial cells by inhibiting the NF-κB and MAPK signaling pathways [31]. RvD1 downregulates NF-κB inflammatory signaling, inhibiting renal cell apoptosis [32]. These studies suggest that RvD1 not only alleviates cellular inflammation but also inhibits cell apoptosis. However, it is currently unclear whether RvD1 can reduce apoptosis in intestinal epithelial cells, especially apoptosis triggered by endoplasmic reticulum stress, despite its ability to reduce small intestinal damage in mice through its anti-inflammatory effect [28].

Therefore, in the present study, we aimed to elucidate the regulatory role of RvD1 in ER stress-induced apoptosis.This was based on the successful establishment of an ER stress model in intestinal porcine epithelial cells (IPEC-J2) using tunicamycin.

## Results

### The stimulation concentration and time of tunicamycin inducing ER stress

**Fig. 1:**
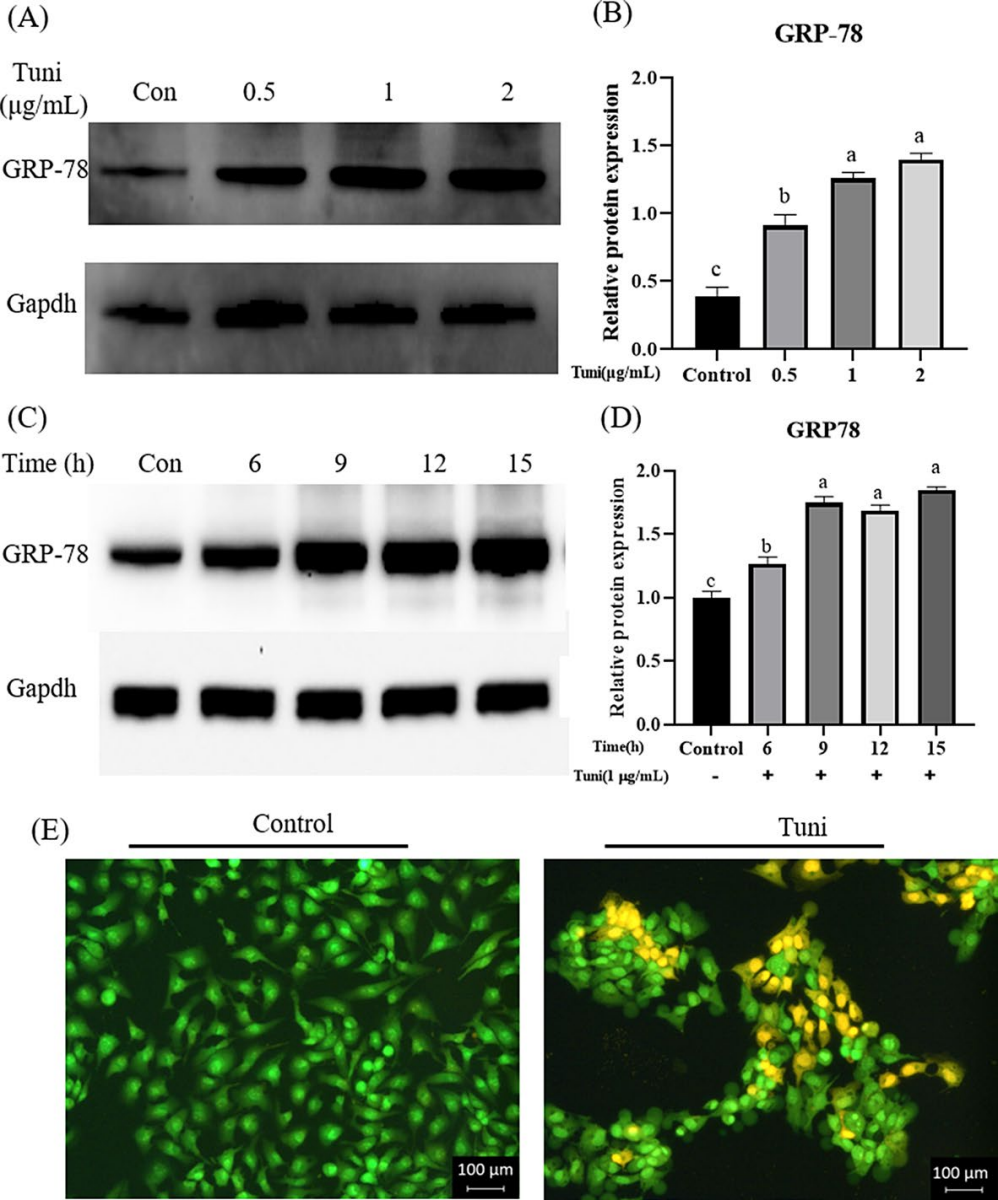
Determination of the stimulation concentration and time of tunicamycin inducing ER stress. (**A**) The protein expression of GRP-78 and GAPDH when the IPEC-J2 cells were stimulated for 6 h with different concentrations of tunicamycin (0, 0.5, 1, and 2 µg/mL). The blots were cut prior to hybridization with antibodies in order to focus on the specific protein bands of interest. As a result, we do not have full-length images of the membranes. However, the cropped images accurately reflect the expression of the target protein. (**B**) The relative gray value of GRP-78 when the IPEC-J2 cells were stimulated for 6 h with different concentrations of tunicamycin (0, 0.5, 1, and 2 µg/mL). (**C**) The protein expression of GRP-78 and GAPDH when the IPEC-J2 cells were stimulated for different durations (0, 6, 9, 12, and 15 h) with 1 µg/mL tunicamycin. (**D**) The relative gray value of GRP-78 when the IPEC-J2 cells were stimulated for different durations (0, 6, 9, 12, and 15 h) with 1 µg/mL tunicamycin. (**E**) Apoptosis presentation using AO/EB fluorescent staining when the cells were stimulated for 9 h with 1 µg/mL tunicamycin to build the ER stress model. The cell morphology was photographed with an inverted microscope (×200). The viable cells appeared uniformly green, the apoptotic cells showed bright green in the nuclei as chromatin condensation and nuclear fragmentation, and the necrotic cells presented bright orange. Different lowercase letters on the graph bars indicate statistically significant differences among the groups (ANOVA with Duncan’s test, *p* < 0.05). Data are presented as means ± SEM. The blots were cropped. The samples derived from the same experiment and that blots were processed in parallel. Tuni, tunicamycin; GRP-78, glucose-regulated protein 78; GAPDH, glyceraldehyde-3-phosphate dehydrogenase, an internal reference protein

The IPEC-J2 cells were treated with varying concentrations of tunicamycin (0, 0.5, 1, and 2 µg/mL) for 6 h. As shown in Fig. 1A and B, tunicamycin concentrations at 0.5, 1, and 2 µg/mL significantly increased the protein expression of GRP-78, an ER stress symbol gene, compared with the control group (*p* = 0.0000, 0.0000, 0.0000). The protein expression of GRP-78 in the 1 µg/mL tunicamycin group was significantly higher than that in the 0.5 µg/mL tunicamycin group(*p*= 0.0016), but there was no significant difference compared with the 2 µg/mL tunicamycin group. Thus, the 1 µg/mL tunicamycin was used for subsequent experiment.

Next, the IPEC-J2 cells were stimulated with 1 µg/mL tunicamycin for different durations (0, 6, 9, 12, and 15 h). Compared with the control group, the protein expression of GRP-78 significantly increased in alltunicamycin treatment groups (*p*= 0.0031, 0.0000, 0.0000, 0.0000) (Fig. 1C and D). The protein expression of GRP-78 at 9 h of stimulation was significantly higher than that at 6 h (p = 0.0000), but there were no significant differences with 12 and 15 h of stimulation (Fig. 1C and D). Based on these results, the cells were treated with 1 µg/mL tunicamycin for 9 h to establish the ER stress model. Under these ER stress-inducing conditions, the cells exhibited significant apoptosis, as identified by morphology after AO/EB staining (Fig. 1E).

### RvD1 cytotoxicity and its effects on cell viability

**Fig. 2:**
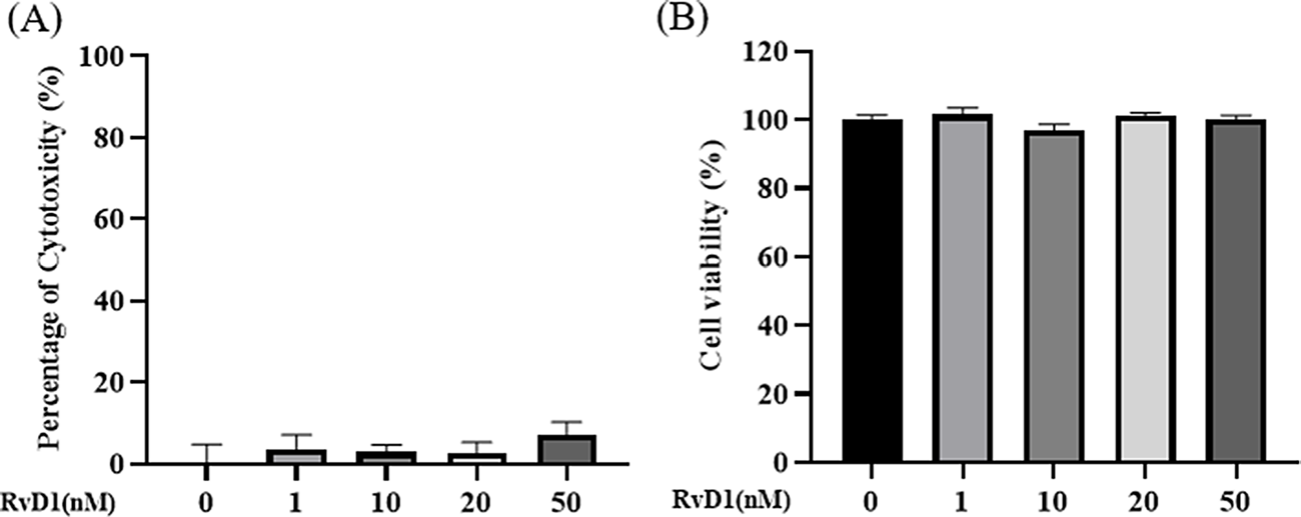
RvD1 cytotoxicity and its effects on cell viability. (**A**) RvD1 cytotoxicity test. (**B**) The effects of RvD1 on cell viability. Without a lowercase letter on the graph bars indicates no significant differences among the groups (ANOVA with Duncan’s test). Data are presented as means ± SEM. RvD1, Resolvin D1

The IPEC-J2 cells were treated with different concentrations of RvD1 (0, 1, 10, 20, and 50 nM) for 24 h to determine the cytotoxicity of RvD1 and its effects on cell viability. As shown in Fig. 2, none of the RvD1 concentrations had a significant cytotoxic effect or influence on cell viability in IPEC-J2 cells.

### The effective concentration of RvD1 relieving ER stress and apoptosis

**Fig. 3:**
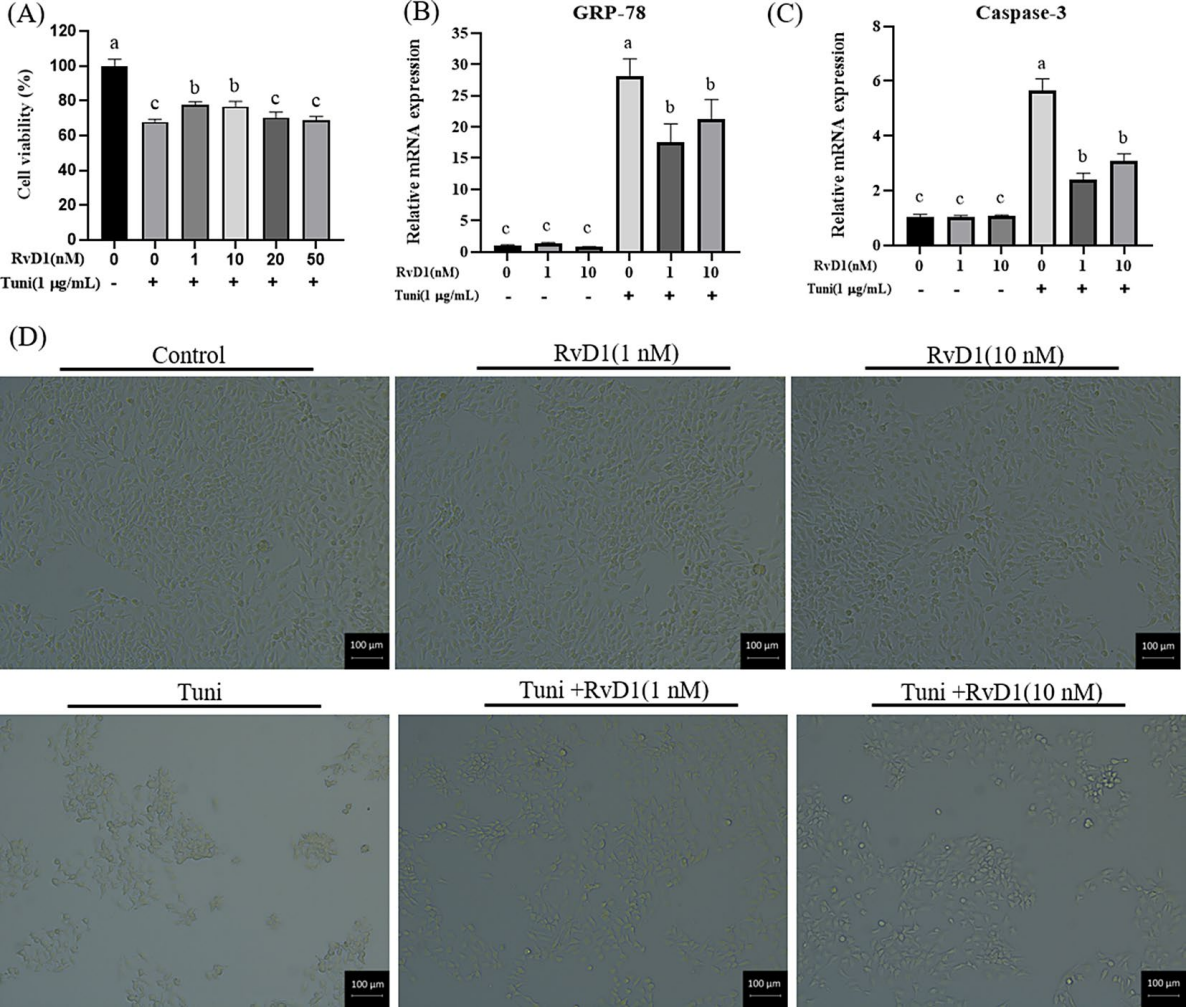
Determination of the effective concentration of RvD1 relieving ER stress and apoptosis. (**A**) The effects of various concentrations of RvD1 (0, 1, 10, 20, 50 nM) on cell viability. (**B**) The effects of various concentrations of RvD1 (0, 1, 10, 20, 50 nM) on the gene expression of GRP-78. (**C**) The effects of 1 and 10 nM RvD1 on the gene expression of Caspase-3. (**D**) The effects of 1 and 10 nM RvD1 on cell morphology. The cell morphology was photographed with an inverted microscope (×100). Different lowercase letters on the graph bars indicate statistically significant differences among the groups (ANOVA with Duncan’s test, *p* < 0.05). Data are presented as means ± SEM. The analysis of gene expression and cell viability are 3 and 8 replicates in each group, respectively. RvD1, Resolvin D1; Tuni, tunicamycin; GRP-78, glucose-regulated protein 78

The IPEC-J2 cells were pre-treated for 15 h using different concentrations of RvD1 (0, 1, 10, 20, and 50 nM). After this time, the cells were further treated for 9 h with the RvD1 and 1 µg/mL tunicamycin. As shown in Fig. 3A, compared with the control group, the cell viability significantly decreased in all the tunicamycin stimulation groups (*p*= 0.0000, 0.0000, 0.0000, 0.0000, 0.0000). Compared with the sole tunicamycin stimulation, cell viability significantly enhanced with 1 and 10 nM RvD1 (*p*= 0.0154, 0.0202).

As shown in Fig. 3B C, compared with the control group, tunicamycin stimulation significantly increased the gene expression *GRP-78* (*p*= 0.0000, 0.0001, 0.0000) and *Caspase-3* (*p*= 0.0000, 0.0011, 0.0000). Compared with the sole tunicamycin stimulation,  1 nM RvD1 significantly decreased the gene expression of *GRP-78* (*p*= 0.0037, 0.0353) and *Caspase-3* (*p*= 0.0000, 0.0000). The 1 nM RvD1 treatment resulted in a greater decrease in the gene expression of *GRP-78* and *Caspase-3* than the 10 nM RvD1 treatment, but the differences were not significant. The changes of cell morphology further confirmed above results. The sole tunicamycin stimulation caused cell shrinkage and decreased the number of the cells, but the 1 and 10 nM RvD1 reduced the negative effects to a certain extent (Fig. 3D). Combining the results in Fig. 3, the 1 nM RvD1 was selected to treat cells for relieving ER stress and associated apoptosis.

### RvD1 reduced apoptosis induced by ER stress

**Fig. 4:**
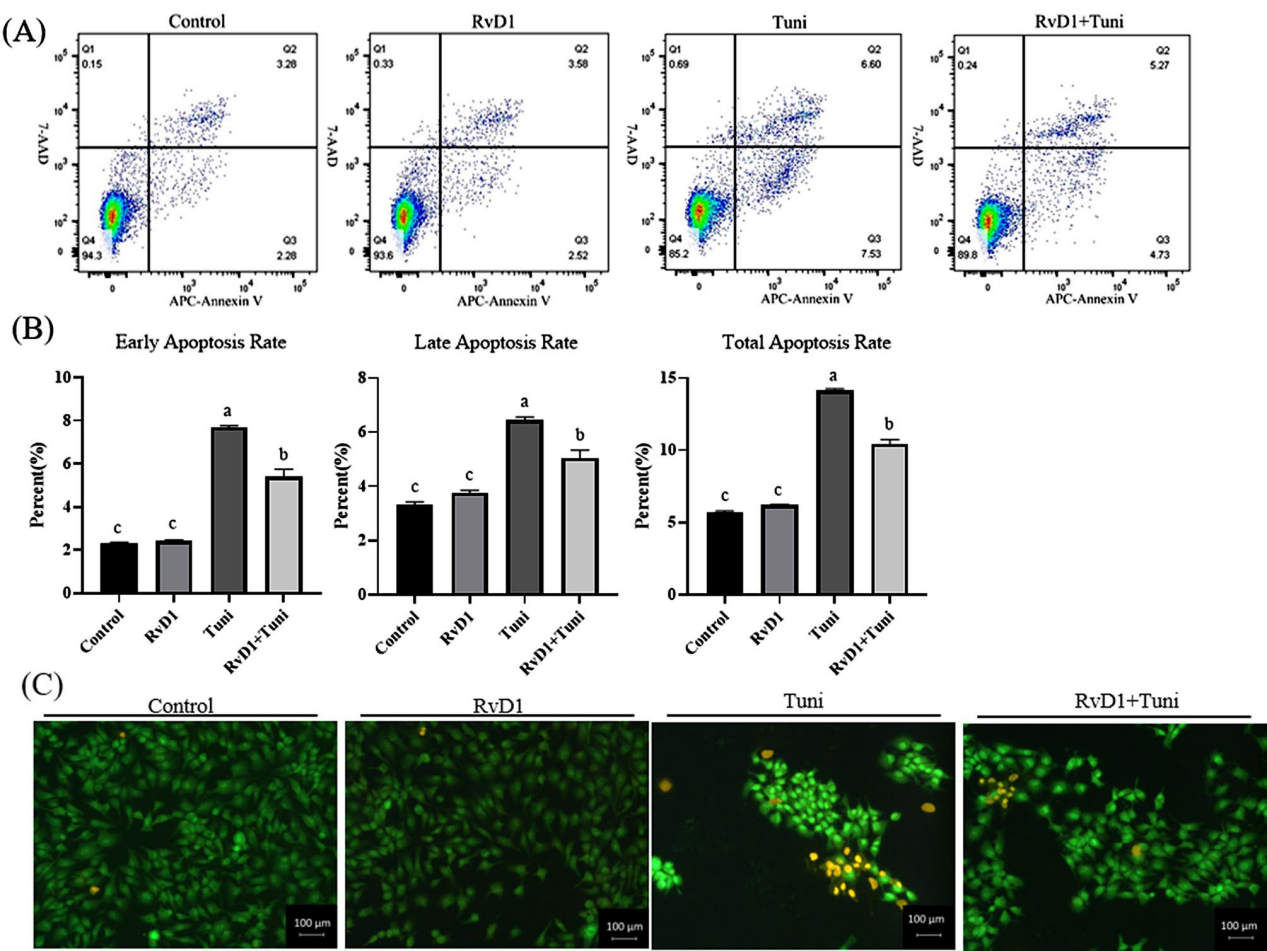
RvD1 reduced apoptosis induced by tunicamycin. After stimulation, the cells were stained with Annexin V-APC/7-AAD for flow cytometry analysis and with AO/EB for morphological assessment. (**A**) Apoptosis determination using flow cytometry. The Q1, Q2, Q3, and Q4 in the flow cytometry images indicated cell debris, late apoptotic cells, early apoptotic cells, and viable cells, respectively. (**B**) Apoptosis rate analysis based on flow cytometry. (**C**) Apoptosis presentation using AO/EB fluorescent staining. The cell morphology was photographed with an inverted microscope (×200). The viable cells appeared uniformly green, the apoptotic cells showed bright green in the nuclei as chromatin condensation and nuclear fragmentation, and the necrotic cells presented bright orange. Different lowercase letters on the graph bars indicate statistically significant differences among the groups (ANOVA with Duncan’s test, *p* < 0.05). Data are presented as means ± SEM. RvD1, Resolvin D1; Tuni, tunicamycin; Tuni + RvD1, co-treatment with RvD1 and tunicamycin

As shown in Fig. 4A and B, flow cytometry analysis showed that the cell apoptosis rate (early, late, and total) in the tunicamycin  (*p*= 0.0000, 0.0000, 0.0000) and tunicamycin + RvD1 (*p*= 0.0000, 0.0001, 0.0000) groups was significantly higher than that in the control groups. Compared with the tunicamycin group, the apoptosis rate (early, late, and total) significantly decreased in the tunicamycin + RvD1 group  (*p*= 0.0000, 0.0005, 0.0000). To further determine the effect which RvD1 decreased the apoptosis induced by tunicamycin, the apoptotic cells were identified by morphology after AO/EB staining. After dyeing, the viable cells appeared uniformly green, the apoptotic cells showed bright green in the nuclei as chromatin condensation and nuclear fragmentation, and the necrotic cells presented bright orange. As shown in Fig. 4C, tunicamycin significantly promoted apoptosis, but RvD1 effectively alleviated this apoptosis, which further confirmed the results of flow cytometry analysis.

### RvD1 decreased the expression of apoptosis and ER stress genes

**Fig. 5:**
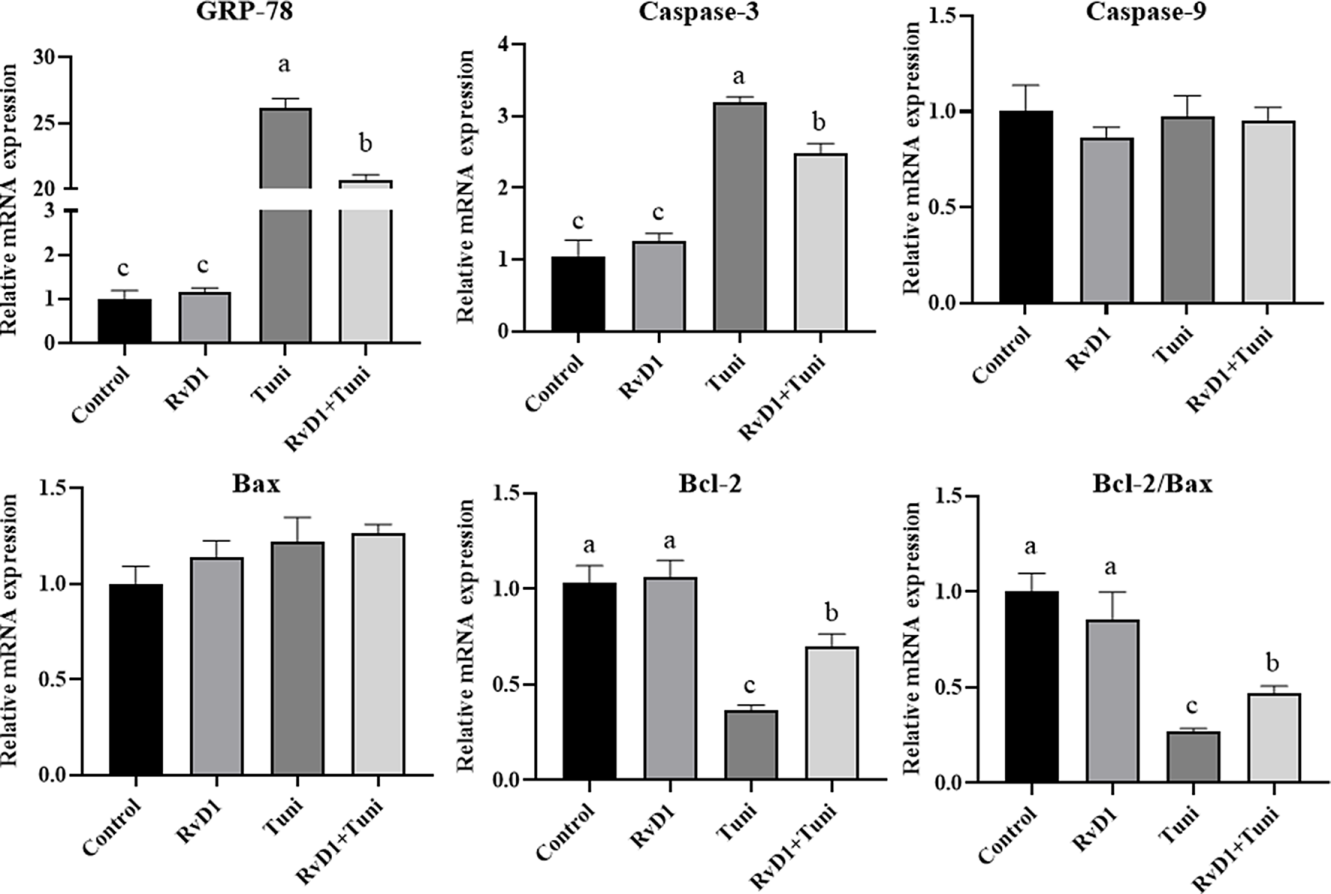
The mRNA expression of apoptosis and endoplasmic reticulum (ER) stress genes. Different lowercase letters on the graph bars indicate statistically significant differences among the groups (ANOVA with Duncan’s test, *p* < 0.05). Data are presented as means ± SEM. RvD1, Resolvin D1; Tuni, tunicamycin; Tuni + RvD1, co-treatment with RvD1 and tunicamycin; *GRP-78*, glucose-regulated protein 78; *Bcl-2*, B cell lymphoma 2; *Bax*, *Bcl-2*-associated X protein

As shown in Fig. 5, compared with the control groups, the gene expression of GRP-78 (*p*= 0.0000, 0.0000) and Caspase-3 (*p*= 0.0000, 0.0001) significantly increased in the tunicamycin and tunicamycin + RvD1 groups. Co-treatment with tunicamycin and RvD1 significantly decreased the gene expression of *GRP-78* (*p*= 0.0000) and *Caspase-3* (*p*= 0.0095), compared with the sole tunicamycin stimulation. Although the gene expression of *Bax* and *Caspase-9* was not significant differences among the groups, tunicamycin and tunicamycin + RvD1 significantly decreased the gene expression of *Bcl-2* (*p*= 0.0000, 0.0050) and the ratio of *Bcl-2/Bax* (*p*= 0.0050, 0.0126), compared with the control groups. Tunicamycin + RvD1 significantly increased the gene expression of *Bcl-2* (*p*= 0.0000) and the ratio of *Bcl-2/Bax* (*p*= 0.0165), compared with the tunicamycin group.

**Fig. 6:**
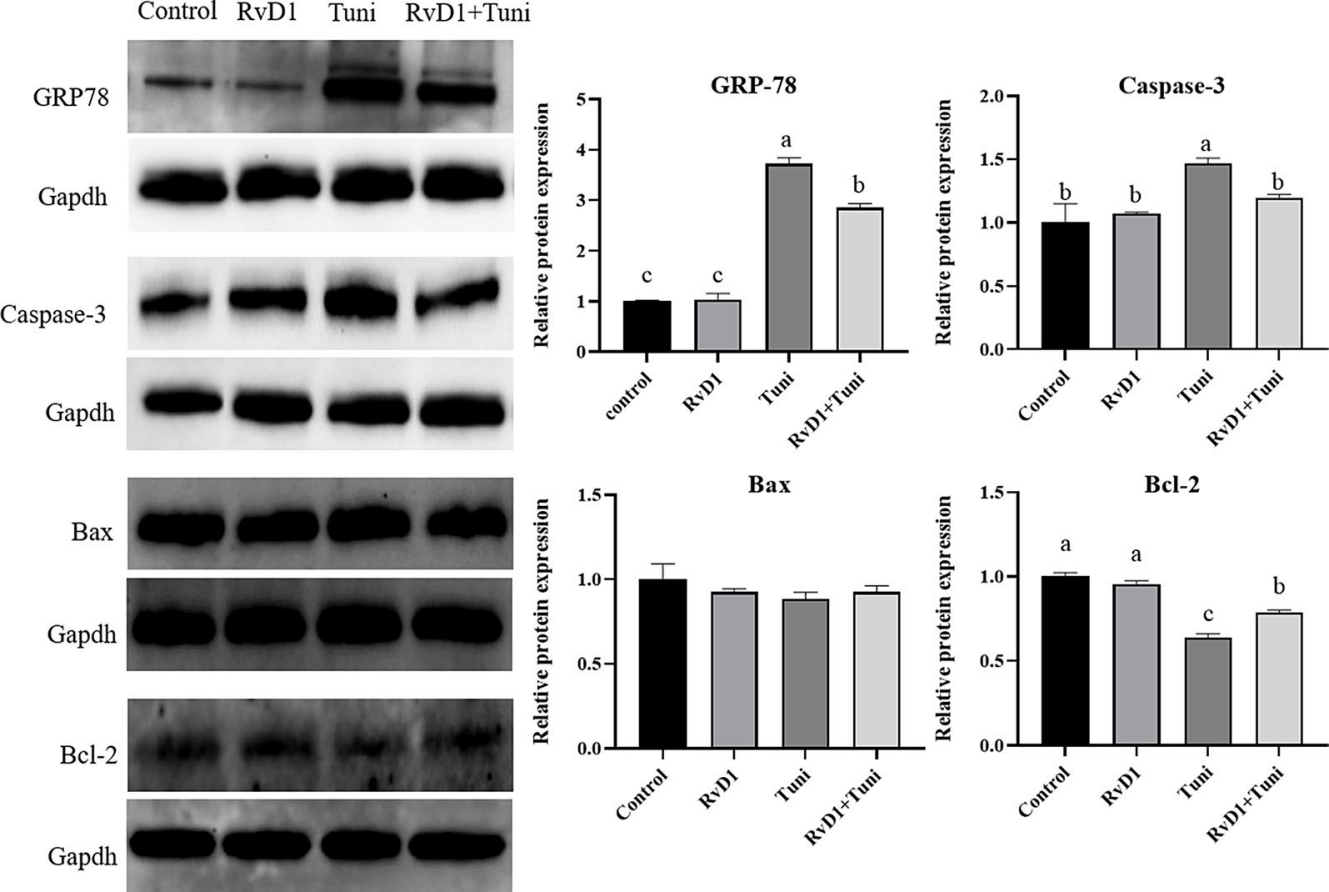
The protein expression of apoptosis and endoplasmic reticulum (ER) stress genes. The blots were cut prior to hybridization with antibodies in order to focus on the specific protein bands of interest. As a result, we do not have full-length images of the membranes. However, the cropped images accurately reflect the expression of the target protein. Different lowercase letters on the graph bars indicate statistically significant differences among the groups (ANOVA with Duncan’s test, *p* < 0.05). Data are presented as means ± SEM. The blots were cropped. The samples derived from the same experiment and that blots were processed in parallel. RvD1, Resolvin D1; Tuni, tunicamycin; Tuni + RvD1: co-treatment with RvD1 and tunicamycin; GRP-78, glucose-regulated protein 78; Bcl-2, B cell lymphoma 2; Bax, Bcl-2-associated X protein; GAPDH: glyceraldehyde-3-phosphate dehydrogenase, an internal reference protein

As shown in Fig. 6, the protein expression of GRP-78 (*p*= 0.0000) and Caspase-3 (*p*= 0.0026) significantly increased in the tunicamycin group, compared with the control groups. Tunicamycin + RvD1 significantly decreased the protein expression of GRP-78 (*p*= 0.0000) and Caspase-3 (*p*= 0.0368), compared with the tunicamycin group. Although the protein expression of Bax was not significantly different among the groups, Tunicamycin and tunicamycin + RvD1 significantly decreased the protein expression of Bcl-2 (*p*= 0.0000, 0.0001) compared with the control groups. Besides, tunicamycin + RvD1 significantly increased the protein expression of Bcl-2 (*p*= 0.0008), compared with the sole tunicamycin treatment.

## Discussion

Tunicamycin, a kind of nucleoside antibiotic, is commonly used as a specific drug for inducing ER stress [33–35]. ER stress has been induced by tunicamycin in the concentration range of 1-5 μg/mL and treatment time range of 4-24 h In HepG2, adipocytes (3T3-L1), HeLa, and other cell types [33–39]. Based on these previous studies, the IPEC-J2 cells in this study were stimulated with tunicamycin for 5 time periods (0, 6, 9, 12, and 15 h) and 4 concentration gradients (0, 0.5, 1, and 2 µg/mL). Our research indicated that the ER stress mode of IPEC-J2 cells could be successfully built by stimulating the cells for 9 h with 1 µg/mL tunicamycin. In a study by Wen et al. [40], they stimulated mammary epithelial cell line, MCF-7 cells, for 24 h with tunicamycin at four different concentrations (0, 0.1, 0.5, and 1 µg/mL), finding that tunicamycin concentrations of 0.5 and 1 µg/mL significantly increased the expression of GRP-78 and caused ER stress, and they finally selected 1 µg/mL tunicamycin to build ER stress model for their follow-up experiment. Similarly, Yang et al. [41]. treated human umbilical vein endothelial cells for 24 h with tunicamycin at five different concentrations (0, 0.25, 0.5, 1, and 2 µg/mL), finding that all concentrations (except concentration = 0) of tunicamycin significantly increased the expression of GRP-78 and caused ER stress, and the expression of GRP-78 was not a significant difference among the three concentrations (0.5, 1, and 2 µg/mL). Finally, they also chose 1 µg/mL tunicamycin to build ER stress model for their follow-up experiment. For IPEC-J2 cells, 1 µg/mL tunicamycin stimulated them for 24 h, which increased the expression of GRP-78 and successfully built ER stress model [33]. Here, our results indicated that three different concentrations (0.5, 1, 2 µg/mL) of tunicamycin significantly increased the expression of GRP-78, but the expression differences were not significant between 1 and 2 µg/mL tunicamycin stimulation, thus 1 µg/mL tunicamycin was used for the follow-up experiments. Our results corroborated the findings reported in previous those studies [33, 40, 41].

The distinction lies in the stimulation time of tunicamycin. Previous research did not screen the stimulation time, but usually adopts a fix-time 24 h [33, 40, 41]. To successfully establish the ER stress model of IPEC-J2 cells, in this study, we not only confirmed a suitable stimulation dose, but also determined the optimal stimulation time of tunicamycin. The protein expression of GRP-78 at stimulation 9 h of stimulation was significantly higher than that at stimulation 6 h and was not significantly differences with that at stimulation 12 and 15 h of stimulation. Therefore, the IPEC-J2 cells were stimulated for 9 h to establish ER stress model for the follow-up experiments in the current study.

RvD1, a bioactive pro-resolving lipid mediator, usually dampens pathological inflammatory responses [42]. It did not induce cytotoxicity in human osteoblastic osteosarcoma cell line MG-63 cells at concentrations of 50, 100, and 200 nM [43]. Besides, it also had no cytotoxic effects on human gingival fibroblasts at concentrations between 1 and 1,000 nM [44]. Furtherly, it was not toxic up to 10 µM in human osteoarthritis chondrocytes [45]. In this study, all the stimulation concentrations (0, 1, 10, 20, and 50 nM) of RvD1 were far below those in previous findings and had no cytotoxicity in IPEC-J2 cells, which was similar to previous research [46]. Meanwhile, except for no cytotoxicity, we also revealed that RvD1 did not affect cell viability of normal IPEC-J2 cells but reduced tunicamycin induced IPEC-J2 cell viability inhibition at the stimulation concentrations of 1 and 10 nM. Cao et al. [43] found that RvD1 (50, 100, and 200 nM) not only had no effects on the viability of MG-63 cells, but repressed LPS induced proliferation inhibition of the cells. In a study by Xu et al. [47], their results indicated that RvD1 (100 and 200 nM) significantly rescued 1-methyl-4-phenylpyridinium ion (Mpp+) induced viability downregulation of pheochromocytoma (PC12) cells. These observations also supported our results in this study.

In addition to the effects of RvD1 on cytotoxicity and viability in IPEC-J2 cells, it also attenuated apoptosis triggered by ER stress in this study. The ER stress model of IPEC-J2 cells was successfully built by stimulating the cells for 9 h with 1 µg/mL tunicamycin, which has been discussed above. Tunicamycin induced ER stress, which in turn triggers apoptosis that has been reported in many studies [15–17]. The apoptosis triggered by ER stress has one thing in common: the expression of GRP-78 and pro-apoptotic protein Bax and caspase-3 is increased, but the expression of anti-apoptotic protein Bcl-2 and the ratio of Bcl-2/Bax are decreased [48–50]. In IPEC-J2 cells, ER stress induced by tunicamycin also increased the expression of GRP-78 and Caspase-3 that caused apoptosis [33]. In this study, the test results of flow cytometry and AO/EB staining showed that tunicamycin increased the apoptosis rate of IPEC-J2 cells, but RvD1 inhibited the pro-apoptotic effect. Furthermore, tunicamycin increased the gene and protein expression of Caspase-3 and GRP-78 and decreased the expression of anti-apoptotic protein Bcl-2, but RvD1 mitigated the adverse impacts. Here, our results not only confirmed the pro-apoptotic effect of ER stress, but also indicated the anti-apoptotic influence of RvD1 in IPEC-J2 cells. GRP-78 as an upstream protein of ER stress activates the downstream expression of CHOP and caspase-3 to initiate apoptosis. In this study, RvD1 decreased the gene and protein expression of GRP-78 and Caspase-3 and improved the expression of Bcl-2. It is reasonable to speculate that GRP-78/Caspase-3 pathways involved in the protective effects of RvD1 against ER stress-induced apoptosis.Jung et al. [26]. found that RvD1 (2, 5, and 10 nM) attenuated apoptosis and the expression of Caspase-3 in HepG2 cells. RvD1 concentration of 10 ng/mL suppressed lipopolysaccharide (LPS) caused apoptosis increase and the expression up-regulation of cleaved-Caspase-3 in renal cells [32]. RvD1 (50 nM) inhibited macrophages from oxidative stress-induced apoptosis and promoted the expression of anti-apoptotic protein Bcl-2 and Bcl-xL [51]. Furthermore, RvD1 also rescued the apoptosis of cardiomyocytes [31], retinal cells [52], and renal tissues [53]. These findings combine to further demonstrate that RvD1 reduces ER stress-induced apoptosis in IPEC-J2 cells.

## Conclusion

Stimulation with tunicamycin (1 µg/mL) for 9 h successfully establishes an ER stress model and then induces apoptosis in IPEC-J2 cells. RvD1 has no cytotoxic effects and alleviates apoptosis and viability inhibition through attenuating ER stress and regulating apoptosis-related protein (Caspase-3, Bcl, and Bax) expression in the cells.

## Methods

### Cell culture

IPEC-J2 cells are a kind of porcine intestinal columnar epithelial cell derived from the mid-jejunum of piglets [54]. The cell line used in the experiment was kindly donated from the laboratory of the Academy of Animal Science, China Agricultural University. The cells were cultured in Dulbecco’s Modified Eagle’s Medium Nutrient Mixture F-12 (DMEM/F-12; Gibco, Beijing, China) supplemented with 10% fetal bovine serum (FBS; Gibco, Waltham, MA, USA), 1% 100×ITS liquid media supplement (Sigma-Aldrich, St. Louis, MO, USA), and 1% penicillin/streptomycin (Life Technologies, Carlsbad, CA, USA) in a humidified incubator at 37% with 5% CO_2_. The cells at two to three passages were used for the following experiments.

### Cell viability and cytotoxicity assay

The cells were seeded in a 96-well plate at a density of 8,000 cells per well. At 24 h after culture, the cells grew approximately to 50–60% confluence, and then they were treated for 24 h with different concentrations of RvD1 (0, 1, 10, 20, and 50 nM) for analyzing the effects of RvD1 on cell viability and cytotoxicity. Besides, the cells were pre-treated for 15 h with different concentrations of RvD1 (0, 1, 10, 20, and 50 nM) and then co-treated for 9 h with 1 µg/mL tunicamycin and the pre-treatment concentrations of RvD1 to determine the effects of tunicamycin and RvD1 on cell viability/proliferation. Cell viability was measured using the enhanced Cell Counting Kit-8 (CCK-8) kit (Beyotime, Shanghai, China). After stimulation, 10 µL of CCK-8 solution was added to each treatment well and incubated for 2 h. The optical density (OD) value was detected using a multifunctional microplate reader (BioTek, Winooski, VT, USA) at absorbance 450 nm. Cytotoxicity was analyzed using the lactate dehydrogenase (LDH) cytotoxicity assay kit (Beyotime, Shanghai, China). After stimulation, the 96-well plate was centrifuged to remove the supernatant, and 150 µL of LDH release solution (10-fold dilution) was added to each treatment well and incubated for 1 h. Next, the plate was centrifuged again to aspirate the supernatant 120 µL into a new 96-well plate, and 60 µL of LDH working fluids were add into the supernatant of each well in the new plate. The OD value of absorbance was measured at a wavelength of 490 nm.

### Flow cytometry/annexin V-APC/7-AAD staining

The cells were seeded in 6-well plates at a density of 2.5 × 10^5^ cells per well with 2 mL of the normal medium. When the cells grew to 50–60% confluence, they were divided into 4 groups (control group, RvD1 group, Tuni group, and RvD1 + Tuni group) with 3 repetitions per group. Their treatments contain two stages. At the first stage, the control and Tuni groups of cells were cultured for 15 h with normal medium, and the RvD1 and RvD1 + Tuni groups of cells were cultured for 15 h with normal medium containing 1 nM RvD1. At the second stage, the old medium in the control, RvD1, Tuni, and RvD1 + Tuni groups was replaced using fresh medium, fresh medium containing 1 nM RvD1, fresh medium containing 1 µg/mL tunicamycin, and fresh medium containing both 1 µg/mL tunicamycin and 1 nM RvD1, respectively. After replacing with the fresh medium, all the 4 groups of cells were continually cultured for another 9 h. Thus, all the 4 groups of cells were treated for 24 h in total with different stimulation conditions.

At 24 h after treatment, the cells were washed twice with PBS and then trypsinized. Next, they were centrifuged for 5 min at 300 × g, resuspended in PBS, and centrifuged again. Then, the cells were resuspended in Annexin V binding buffer and incubated for 15 min with 5 µL of Annexin V-APC and 7-AAD at room temperature in the dark. After the incubation, the apoptosis rate was analyzed by flow cytometry (BD Biosciences, Heidelberg, Germany), according to the manufacturer’s instructions. Flow cytometry data analysis was performed using FlowJo 10 software.

### AO/EB staining

For a more intuitive observation of apoptosis, the cells were dyed with an AO/EB staining kit (Sangon Biotech, Shanghai, China). The cells were seeded in a 24-well plate at a density of 7 × 10^4^ cells per well. When the cells grew to 50–60% confluence, they were treated according to the description in flow cytometry analysis. At 24 h after treatment, the cells were washed twice with PBS and incubated for 10 min with 5 µL of AO staining solution and EB staining solution at room temperature in the dark. After the incubation, the fluorescence images of the cells were observed using an inverted fluorescence microscope (Leica, Wetzlar, Germany).

### Total RNA isolation, reverse transcription, and real-time quantitative PCR analysis

After treatment, the total cellular RNAs were isolated using the TRIzol reagent (Invitrogen, Carlsbad, CA, USA), and their concentrations were detected with the NanoDrop-2000 Spectrophotometer (Thermo Scientific, Wilmington, USA). The obtained total RNAs were reverse-transcribed using the HifairR III 1st Strand cDNA Synthesis SuperMix (YESEN, Shanghai, China). The reverse transcription was performed in a ProFlex PCR System (Life Technologies, Grand Island, NY, USA), and its incubation program was indicated below as: “25 ℃ for 5 min, 55 ℃ for 15 min, and 85 ℃ for 5 min”. After reverse transcription, the obtained cDNA samples were amplified using the Hieff UNICONR Universal Blue qPCR SYBR Green Master Mix kit (YESEN, Shanghai, China) and gene primers on an ABI QuantStudio6 Real-Time PCR system (Applied Biosystems, Foster City, USA). The amplification procedure was presented below as: “95 ℃ for 2 min, 40 cycles at 95 ℃ for 10 sec and 60 ℃ for 30 sec”, and a melting curve program (at 95 ℃ for 15 s, 60 ℃ for 60 s, and 95 ℃ for 15 s) was added to its tail to ensure specific amplification. The coding sequences (CDs) of target genes were found in NCBI GeneBank and used for designing specific primers in NCBI primer-BLAST. The designed primers were synthesized and purified by Sangon Biotech Co., Ltd (Shanghai, China). The primer sequences were provided in the supplementary Table S1. The relative expression amounts of mRNA were normalized with the internal reference gene glyceraldehyde-3-phosphate dehydrogenase (GAPDH) and calculated by the classic 2^−ΔΔCT^ method.

### Protein extraction and western blotting analysis

After treatment, the total cellular protein was extracted at 4 °C using radioimmunoprecipitation (RIPA) lysis buffer (Beyotime, Shanghai, China) and protease inhibitor phenylmethanesulfonyl fluoride (PMSF; Beyotime, Shanghai, China) according to the ratio of 100:1, and its concentration was determined using the bi-cinchoninic acid (BCA) protein assay kit (CWBIO, Beijing, China) on a multifunctional microplate reader (BioTek, Winooski, VT, USA). The protein was adjusted to an equal concentration of 1.5 µg/µL using lysis buffer, mixed with 4 × loading buffer (Beyotime, Shanghai, China), and heated at 100 °C for 15 min. A total of 15 µg proteins (10 µL of protein solution) per sample were size-fractionated by SDS-PAGE gel (Life Technologies, Grand Island, NY, USA) and electrotransferred onto a polyvinylidene difluoride (PVDF) membrane (GE Healthcare, Waukesha, WI, USA). After being blocked for 1 h at room temperature with 5% skimmed milk powder (Solarbio, Beijing, China) in Tris-buffered saline with Tween-20 (TBST) buffer, the membrane was incubated overnight at 4 ℃ with primary antibody. Subsequently, the membrane was washed three times for 10 min each with TBST buffer and then incubated for 1 h at room temperature with secondary antibody. After incubation, the membrane was washed three times again for 10 min each with TBST buffer. Finally, immunoreactive bands were visualized with enhanced chemiluminescent (ECL) substrate reagent (Bio-Rad, Hercules, CA, USA) on a ChemiDoc™ Touch Imaging System (Bio-Rad, Hercules, CA, USA). The band intensity was quantified as gray value using ImageJ software (National Institutes of Health, Bethesda, MD, USA), and the relative protein levels were normalized by the internal reference protein GAPDH. The primary antibodies included GRP-78 (66574-1-Ig, 1:1000 dilution; Proteintech, Wuhan, China), Caspase-3 (#9668, 1:800 dilution; CST, Danvers, MA, USA), Bcl-2 (#15,071, 1:800 dilution; CST, Danvers, MA, USA), Bax (#14,796, 1:1000 dilution; CST, Danvers, MA, USA), and GAPDH (#3683, 1:1000 dilution; CST, Danvers, MA, USA). The secondary antibodies included Goat anti-Mouse IgG (#31,430, 1:2000 dilution; ThermoFisher, Waltham, MA, USA) and Goat anti-Rabbit IgG (#31,460, 1:2000 dilution; ThermoFisher, Waltham, MA, USA).

### Statistical analysis

Statistical analysis was carried out using statistics software IBM SPSS version 22.0 (IBM Inc., Armonk, NY, USA). One-way ANOVA followed by Duncan’s multiple range test was performed to determine the statistical differences among the groups. Probability values (p-values) less than 0.05 (*p* < 0.05) indicate statistically significant. The data obtained are presented as means ± standard error of the mean (SEM).

### Electronic supplementary material

Below is the link to the electronic supplementary material.

Supplementary Material 1: The primer sequences of target genes.

Supplementary Material 2: Uncropped protein plots corresponding to Figures 1 and 6.

## Data Availability

The datasets used and/or analyzed during the study are available from the corresponding author on reasonable request.

## List of abbreviations

RvD1: Resolvin D1
DHA: docosahexaenoic acid
ER: endoplasmic reticulum
IPEC-J2: intestinal porcine epithelial cells
UPR: unfolded protein response
GRP-78: glucose-regulated protein 78
Bcl-2: B cell lymphoma 2
Bax: B cell lymphoma 2 (Bcl-2)-associated X protein
Tuni: tunicamycin
