# Supplementary material for: Resolvin D1 alleviates apoptosis triggered by endoplasmic reticulum stress in IPEC-J2 cells
Source: BMC Vet Res. 2024 Apr 1;20:125. doi: 10.1186/s12917-023-03820-z (PMC10983747; doi:10.1186/s12917-023-03820-z)
Supplement: Supplementary file 1 — Supplementary Material 1: The primer sequences of target genes. [file 12917_2023_3820_MOESM1_ESM.docx]

| **Genes** | **Primer sequences (5' to 3')** | **Product size/bp** | **Accession number** |
| --- | --- | --- | --- |
| *GRP-78* | **F:** CGGAGGAGGAGGACAAGAAGGAG  **R:** ATATGACGGCGTGATGCGGTTG | 143 | XP_001927830.4 |
| *Caspase-3* | **F:** TGTGGGATTGAGACGGACAGTGG  **R:** GCCAGGAATAGTAACCAGGTGCTG | 112 | NP_999296.1 |
| *Caspase-9* | **F:** CATTGAGACCCTGGATGGCGTTC  **R:** CCCTTTCACTGAGACAGCATTGGAG | 97 | XP_013854451.1 |
| *Bcl-2* | **F:** CAGAGGGGCTACGAGTGGGATG  **R:** CCGGGCTGGGAGGAGAAGATG | 89 | XP_020955252.1 |
| *Bax* | **F:** ATCGGCTGCTGGGCTGGATC  **R:** ATGGTGAGCGAGGCGGTGAG | 124 | XP_003127338.2 |
| *GAPDH* | **F:** CATCAAGAAGGTGGTGAA  **R:** AAGTGGAAGAGTGAGTGT | 92 | NM-001206359.1 |

**Table S1.** The primer sequences of target genes

*GRP-78*, glucose-regulated protein 78; *Bcl-2*, B cell lymphoma 2; *Bax*, *Bcl-2*-associated X protein; *GAPDH*, glyceraldehyde-3-phosphate dehydrogenase, an internal reference protein.
