# Supplementary material for: Resolvin D1 alleviates apoptosis triggered by endoplasmic reticulum stress in IPEC-J2 cells
Source: BMC Vet Res. 2024 Apr 1;20:125. doi: 10.1186/s12917-023-03820-z (PMC10983747; doi:10.1186/s12917-023-03820-z)
Supplement: Supplementary file 2 — Supplementary Material 2: Uncropped protein plots corresponding to Figures 1 and 6. [file 12917_2023_3820_MOESM2_ESM.pptx]

## Slide 1
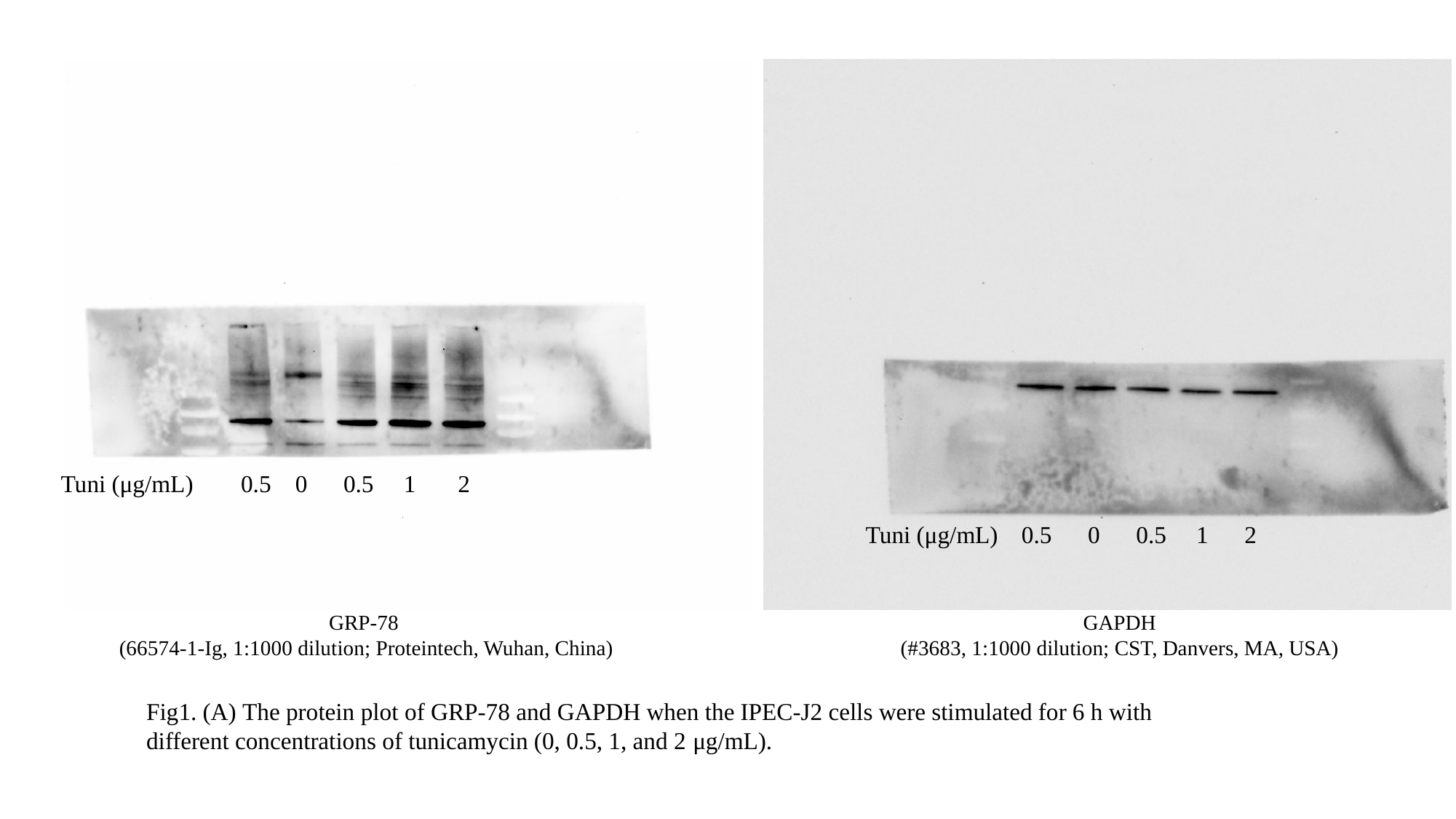

Tuni (μg/mL)
 0.5 0 0.5 1 2
Tuni (μg/mL)
 0.5 0 0.5 1 2
GRP-78
(66574-1-Ig, 1:1000 dilution; Proteintech, Wuhan, China)
GAPDH
(#3683, 1:1000 dilution; CST, Danvers, MA, USA)
Fig1. (A) The protein plot of GRP-78 and GAPDH when the IPEC-J2 cells were stimulated for 6 h with different concentrations of tunicamycin (0, 0.5, 1, and 2 μg/mL).

## Slide 2
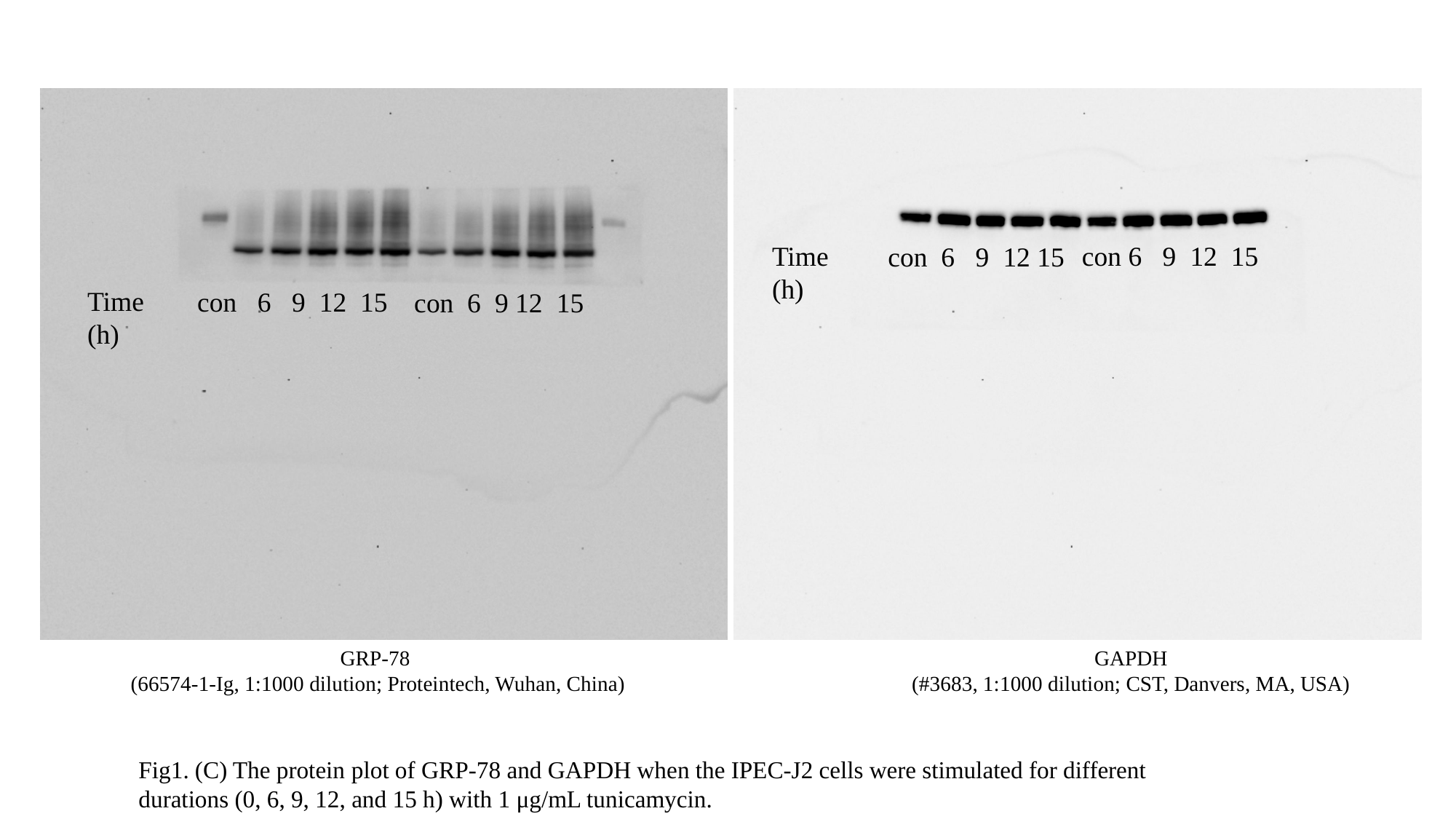

Time (h)
con 6 9 12 15
con 6 9 12 15
Time (h)
con 6 9 12 15
 con 6 9 12 15
GRP-78
(66574-1-Ig, 1:1000 dilution; Proteintech, Wuhan, China)
GAPDH
(#3683, 1:1000 dilution; CST, Danvers, MA, USA)
Fig1. (C) The protein plot of GRP-78 and GAPDH when the IPEC-J2 cells were stimulated for different durations (0, 6, 9, 12, and 15 h) with 1 μg/mL tunicamycin.

## Slide 3
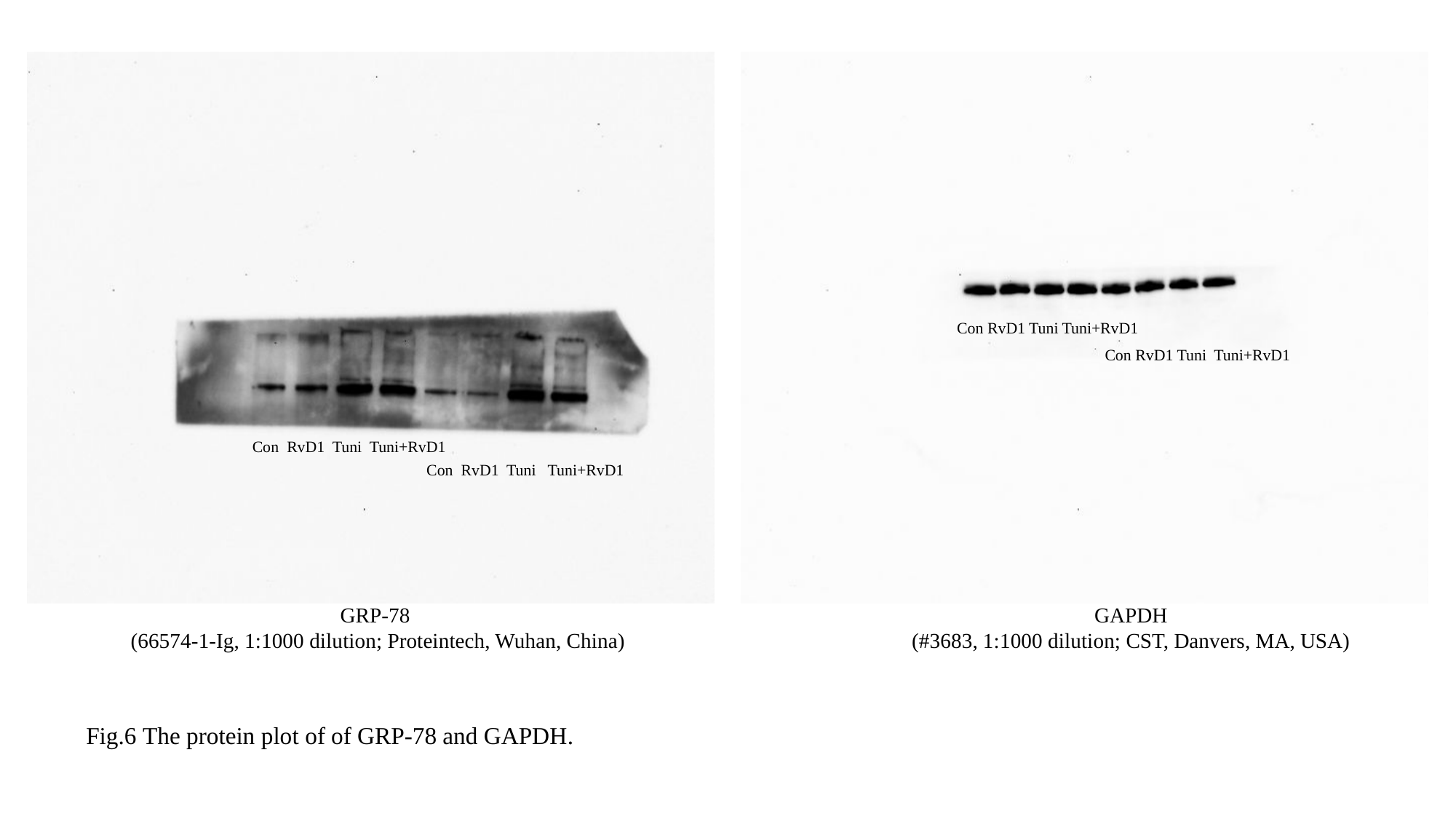

Con RvD1 Tuni Tuni+RvD1
Con RvD1 Tuni Tuni+RvD1
Con RvD1 Tuni Tuni+RvD1
Con RvD1 Tuni Tuni+RvD1
GRP-78
(66574-1-Ig, 1:1000 dilution; Proteintech, Wuhan, China)
GAPDH
(#3683, 1:1000 dilution; CST, Danvers, MA, USA)
Fig.6 The protein plot of of GRP-78 and GAPDH.

## Slide 4
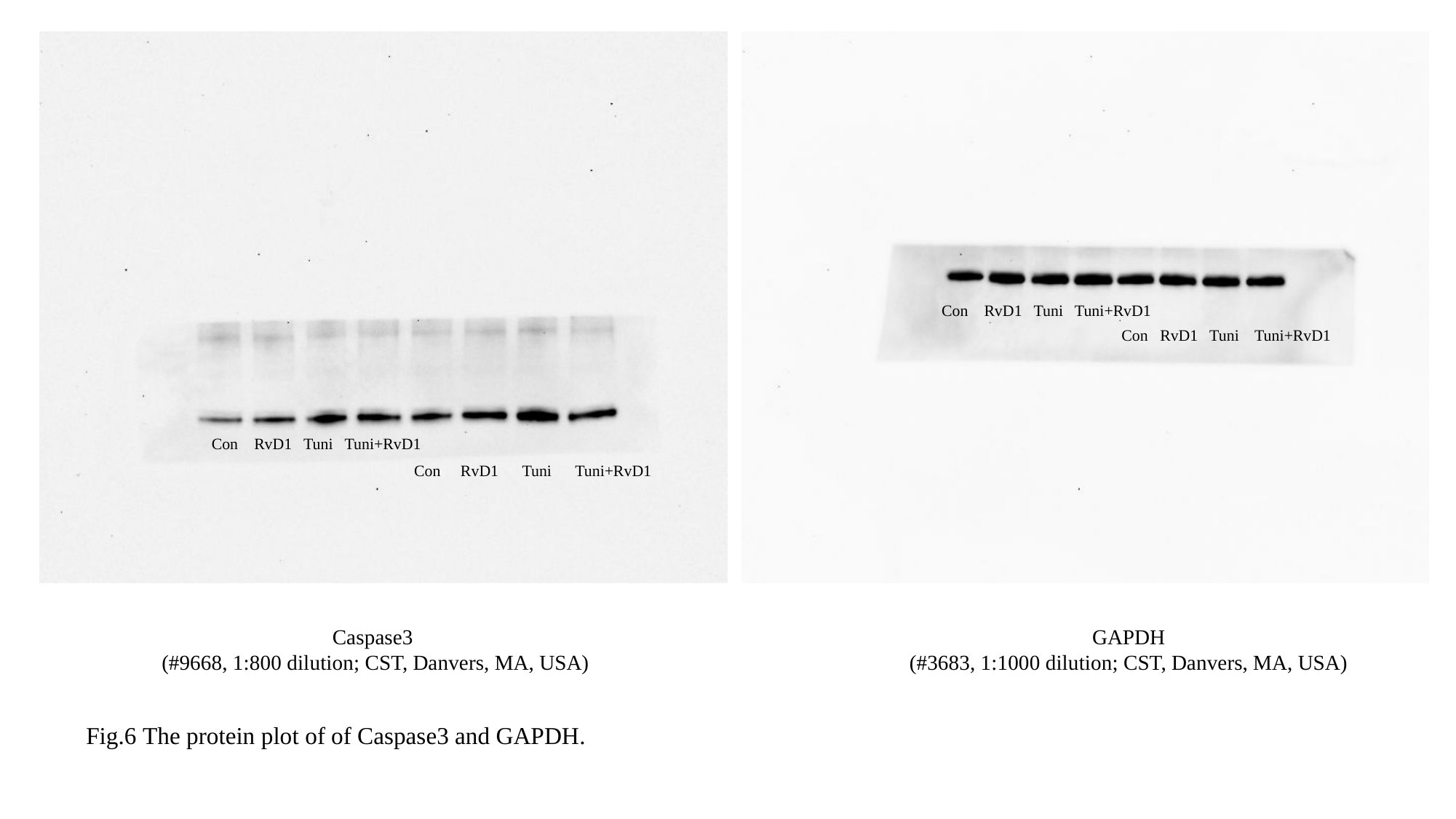

Con RvD1 Tuni Tuni+RvD1
Con RvD1 Tuni Tuni+RvD1
Con RvD1 Tuni Tuni+RvD1
Con RvD1 Tuni Tuni+RvD1
Caspase3
(#9668, 1:800 dilution; CST, Danvers, MA, USA)
GAPDH
(#3683, 1:1000 dilution; CST, Danvers, MA, USA)
Fig.6 The protein plot of of Caspase3 and GAPDH.

## Slide 5
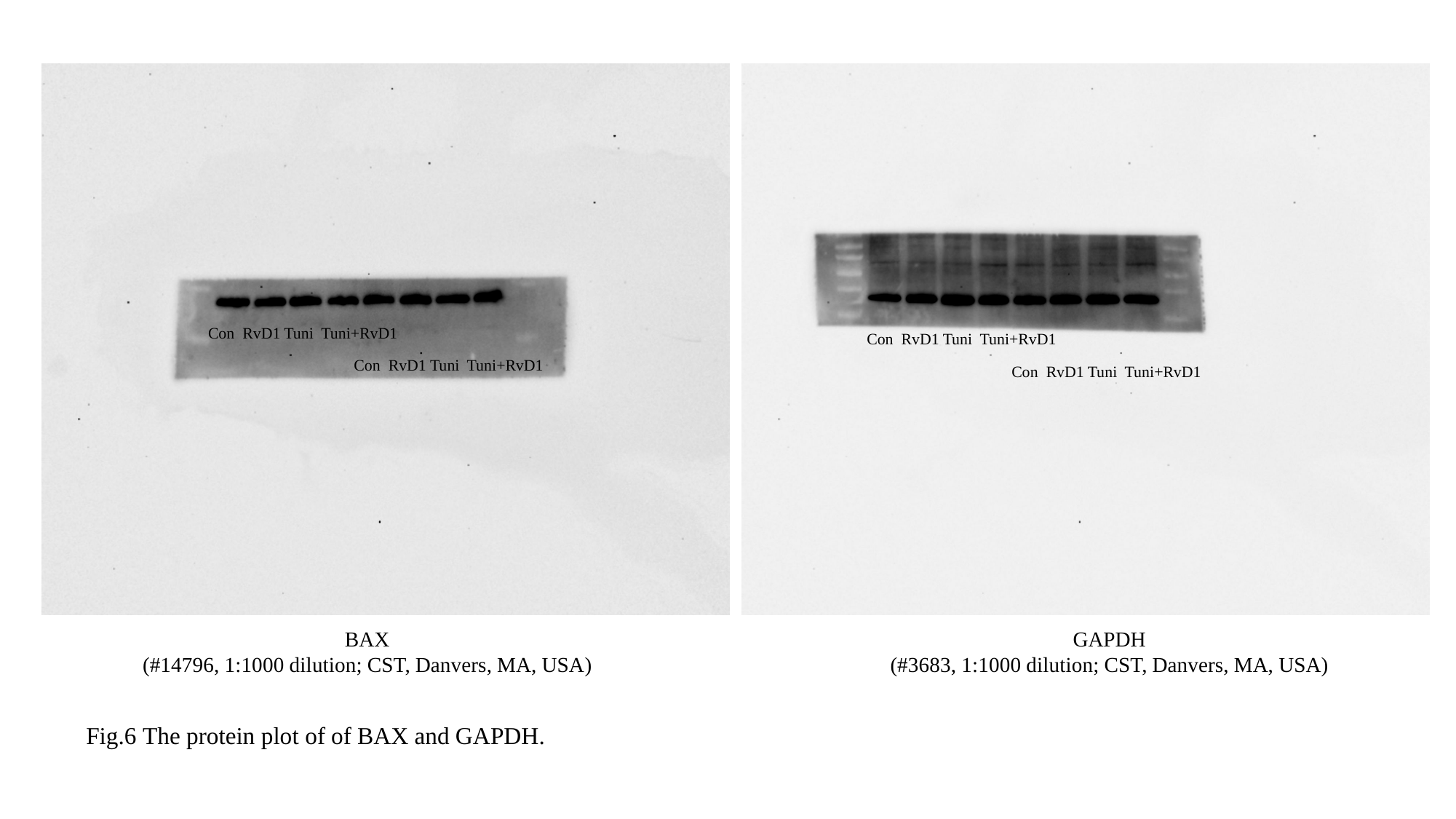

Con RvD1 Tuni Tuni+RvD1
Con RvD1 Tuni Tuni+RvD1
Con RvD1 Tuni Tuni+RvD1
Con RvD1 Tuni Tuni+RvD1
BAX
(#14796, 1:1000 dilution; CST, Danvers, MA, USA)
GAPDH
(#3683, 1:1000 dilution; CST, Danvers, MA, USA)
Fig.6 The protein plot of of BAX and GAPDH.

## Slide 6
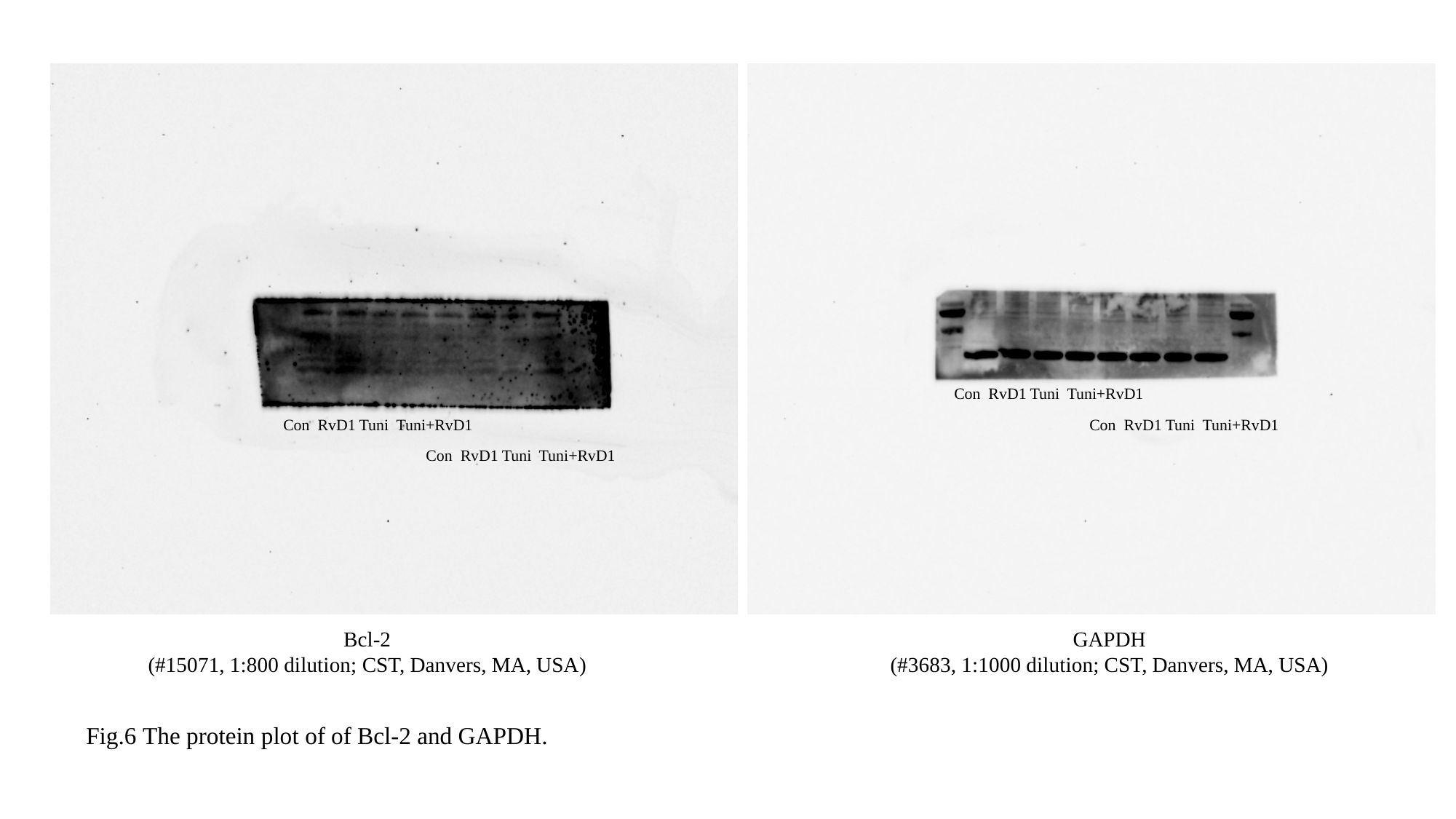

Con RvD1 Tuni Tuni+RvD1
Con RvD1 Tuni Tuni+RvD1
Con RvD1 Tuni Tuni+RvD1
Con RvD1 Tuni Tuni+RvD1
Bcl-2
(#15071, 1:800 dilution; CST, Danvers, MA, USA)
GAPDH
(#3683, 1:1000 dilution; CST, Danvers, MA, USA)
Fig.6 The protein plot of of Bcl-2 and GAPDH.
